# Loss of PIM2 enhances the anti-proliferative effect of the pan-PIM kinase inhibitor AZD1208 in non-Hodgkin lymphomas

**DOI:** 10.1186/s12943-015-0477-z

**Published:** 2015-12-08

**Authors:** S. Kreuz, K. B. Holmes, R. M. Tooze, P. F. Lefevre

**Affiliations:** Section of Experimental Haematology, Leeds Institute of Cancer and Pathology, The Wellcome Trust Brenner Building, St. James’s University Hospital, Leeds, LS9 7TF UK

**Keywords:** PIM kinase, c-MYC, Lymphoma, BL, DLBCL

## Abstract

**Background:**

A promising therapeutic approach for aggressive B-cell Non-Hodgkin lymphoma (NHL), including diffuse large B-cell lymphoma (DLBCL), and Burkitt lymphoma (BL) is to target kinases involved in signal transduction and gene regulation. PIM1/2 serine/threonine kinases are highly expressed in activated B-cell-like DLBCL (ABC-DLBCL) with poor prognosis. In addition, both PIM kinases have a reported synergistic effect with c-MYC in mediating tumour development in several cancers, *c*-*MYC* gene being translocated to one of the immunoglobulin loci in nearly all BLs.

**Methods:**

For these reasons, we tested the efficiency of several PIM kinase inhibitors (AZD1208, SMI4a, PIM1/2 inhibitor VI and Quercetagetin) in preventing proliferation of aggressive NHL-derived cell lines and compared their efficiency with PIM1 and/or PIM2 knockdown.

**Results:**

We observed that most of the anti-proliferative potential of these inhibitors in NHL was due to an off-target effect. Interestingly, we present evidence of a kinase-independent function of PIM2 in regulating cell cycle. Moreover, combining AZD1208 treatment and PIM2 knockdown additively repressed cell proliferation.

**Conclusion:**

Taken together, this study suggests that at least a part of PIM1/2 oncogenic potential could be independent of their kinase activity, justifying the limited anti-tumorigenic outcome of PIM-kinase inhibitors in NHL.

**Electronic supplementary material:**

The online version of this article (doi:10.1186/s12943-015-0477-z) contains supplementary material, which is available to authorized users.

## Background

Diffuse large B cell lymphoma (DLBCL) and Burkitt lymphoma (BL) are aggressive lymphomas, which require intensive chemotherapy regimens. BL is characterised by a germinal centre B-cell phenotype and an isolated c-MYC-rearrangement placing the c-MYC gene into close proximity of one of the Ig enhancers (IgH, Igκ or Igλ) [1]. BL depends on the activity of the transcription factor and proto-oncoprotein c-MYC for proliferation and survival [2, 3] and can be successfully treated with high intensity chemotherapy. DLBCL is significantly more common than BL, accounting for about one third of the NHL cases and is a heterogeneous disease [4]. DLBCL can be classified into two principle classes predictive of disease outcome: germinal-centre B cell-like (GCB), and activated B cell-like (ABC) [5–7]. GCB-DLBCL cells derive from activated germinal centre B cells and about 85 % of the cases express BCL6 a key transcriptional regulator of the GC response [8]. ABC-DLBCL cells are post-germinal centre B cells, with features indicative of arrest during plasmablastic differentiation, and frequently show evidence of NFκB pathway activation [6, 7, 9]. Both ABC-DLBCL and GCB-DLBCL can be associated with c-MYC deregulation or evidence of c-MYC activation. C-MYC-translocations occurring in the presence of a second or third translocation usually affecting BCL2 or BCL6 identify poor risk DLBCL referred to as double or triple hit lymphoma. Deregulation of c-MYC is therefore a common feature amongst aggressive lymphoma either occurring as an isolated event or with additional rearrangements.

Amongst the genes distinguished by differential expression in DLBCL subsets are the PIM (proviral integration site for Moloney murine leukaemia virus) kinases. High *PIM1* and *PIM2* mRNA levels are characteristic of ABC-DLBCL compared to GCB-DLBCL cells [7, 10]. Expression of PIM1 and/or PIM2 is predictive of disease-free, disease-specific and overall survival in non-GCB DLBCL [11, 12] and predominant nuclear PIM1 staining is highly correlated with disease stage in this type [11]. These kinases are of particular interest because of a potential role as co-regulators of c-MYC dependent oncogenesis, with c-Myc and Pim1 showing co-operation during lymphomagenesis in mouse models [13, 14]. Furthermore c-MYC and PIM1 have also been shown to cooperate in prostate tumourigenesis, while the inhibition of PIM kinases in c-MYC-expressing cancers decreases proliferation, survival and tumourigenicity [15, 16].

The related kinases PIM1, PIM2 and PIM3 form the PIM kinase family of constitutively active serine/threonine kinases [17]. *Pim1* and *Pim2* were initially identified as targets for the integration of Moloney murine leukaemia virus (MMLV) in murine T cell lymphoma, indicating that they function as oncogenes [18, 19]. Early studies showed that *PIM1* is overexpressed in 30 % of human lymphoid and myeloid leukaemias, while *PIM2* is overexpressed in AML [20]. Both *PIM1* and *PIM2* mRNAs are highly expressed in CLL, DLBCL and mantle cell lymphoma (MCL), whereas *PIM2* is also overexpressed in follicular lymphoma, MALT lymphoma, nodal marginal zone lymphoma and multiple myeloma [12, 21]. No overexpression of *PIM3* is seen in NHL [12]. Apart from haematopoietic malignancies, *PIM1* and/or *PIM2* are highly expressed in several solid tumours [22–31]. 

Several mechanisms for the oncogenic potential of PIM kinases and for the cooperation between PIM kinases and c-MYC have been described. PIM1 has been shown to be recruited to the chromatin by binding to the MYC MBII (MYC box II) domain and to stimulate transcription elongation through phosphorylation of histone H3 on serine 10 (H3S10p) [32, 33]. The presence of the H3S10p modification is proposed to promote recruitment of 14-3-3 proteins, which serve as adaptors for the acetyl transferase MOF. MOF acetylates H4K16, which is recognised by the bromodomain containing protein 4 (BRD4), an adapter for P-TEFb [33]. PIM1 has been found to be required for the expression of at least 20 % of the c-MYC-induced genes in HEK293 cells [32]. Further, it has been shown that PIM1 overexpression in prostate cancer cell lines enhances the expression of c-MYC target genes [34]. These findings suggest that a central role for PIM kinases in c-MYC-dependent gene regulation may be generalizable to other cell systems. Indeed, PIM1 was described to be nuclear in BL, which would allow for interaction of PIM1 and c-MYC at the chromatin level [35]. Cooperation has also been identified between PIM2 and c-MYC and requires the ability of PIM2 to stimulate the NFκB pathway via activation of the kinase COT. Blocking NFκB in c-MYC and PIM2 overexpressing cells induces apoptosis *in vitro* and inhibits growth in a tumour graft model [36]. Both PIM1 and PIM2 can also directly stabilise c-MYC by phosphorylating Serine 329 [37].

In addition, PIM kinases have several other pro-survival effects. PIM1, PIM2 and PIM3 phosphorylate BAD at S112 and other sites, which leads to binding of 14-3-3 proteins and inhibits its interaction with anti-apoptotic BCL-X_L_ [38, 39]. PIM1 has also been shown to phosphorylate and inhibit the apoptosis signalling kinase 1 (ASK1), which results in reduced JNK and p38 MAPK phosphorylation and protects cells from H_2_O_2_-induced apoptosis [40]. PIM kinases share several substrates with AKT and PIM2 can compensate for mTORC1 inhibition during haematopoiesis and in AML [41, 42]. Additionally, PIM kinases stimulate cell cycle progression by phosphorylating MARK3, CDC25A, CDC25C, p21^CIP1^, p27^KIP1^ and SKP2 [43–49]. PIM1-mediated phosphorylation has also been found to promote the degradation of FOXO1a and FOXO3a, which inhibits FOXO-mediated activation of *CDKN1B* transcription (encoding for p27^KIP1^) [46]. Thus several mechanisms have been identified for c-MYC-independent regulation of proliferation and survival by PIM kinases.

Taken together, these observations provide a rational for targeting PIM1 and PIM2 in c-MYC expressing aggressive lymphomas including ABC-DLBCL and BL, in which PIM kinase inhibition might reduce c-MYC-mediated cell proliferation. In this study, we have assessed the anti-proliferative potential of the pan-PIM kinase inhibitor AZD1208 and other PIM inhibitors in aggressive NHL-derived cell lines and compared it with PIM1 and/ or PIM2 knockdown. Interestingly, our experiments reveal a kinase-independent function of PIM2 in regulating cell cycle. Both AZD1208 treatment and PIM2 knockdown synergistically block cell proliferation, indicating that PIM kinase inhibitors are not sufficient to completely abolish PIM function in NHL.

## Results

### Inhibition of PIM kinases has a minor effect on BL and ABC-DLBCL cell viability

**Fig. 1:**
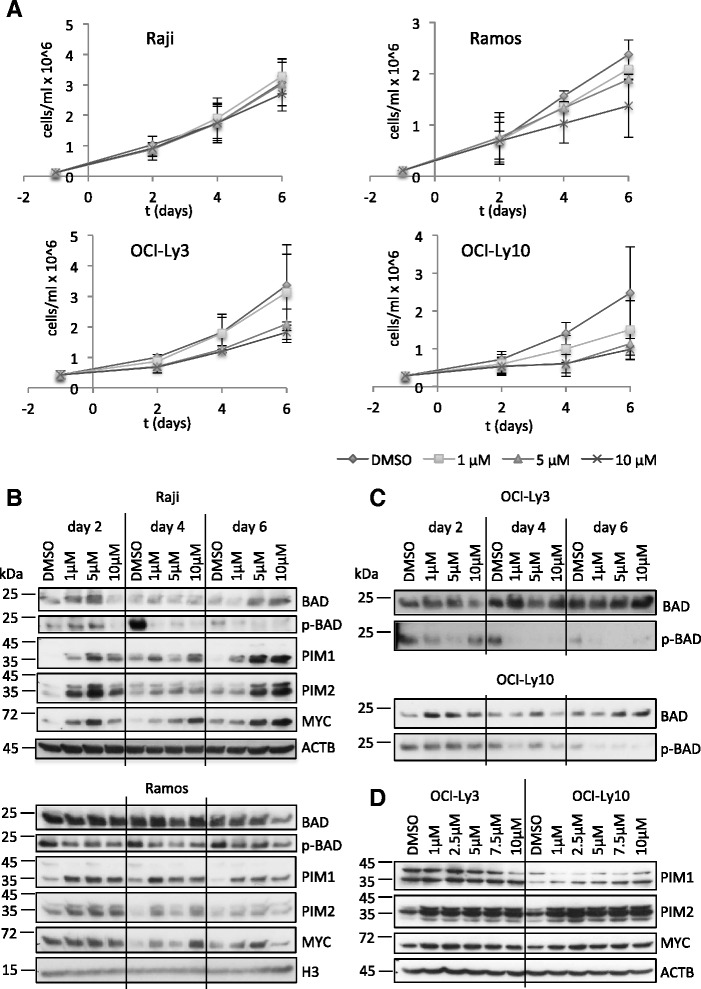
NHL cell lines are resistant to AZD1208 treatment. **a** Cells were seeded into duplicate wells of 6-well plates and treated with DMSO, 1 μM, 5 μM or 10 μM AZD1208 for indicated times. The medium was replaced every day and cell number was measured by MTT assay. The means and SD of at least two independent experiments, conducted in duplicate wells of six-well plates, are plotted. **b**, **c** Cells were treated like in (**a**). Aliquots were harvested every second day and analysed by western blot. **d** Cells were treated with 1 μM, 2.5 μM, 5 μM, 7.5 μM or 10 μM AZD1208 or a respective amount of DMSO for three days. Then, protein expression was evaluated by western blot

The high level of expression of PIM1 and PIM2 in ABC-DLBCL (Additional file 1: Figure S1) and the reported synergistic effect of these two kinases with c-MYC in several cancers, prompted us to test the efficiency of the pan-PIM kinase inhibitor AZD1208 in preventing proliferation of aggressive NHL-derived cell lines. To this end, two BL-derived cell lines, Raji and Ramos, and two ABC-DLBCL-derived cell lines, OCI-Ly3 and OCI-Ly10, were treated with different concentrations of AZD1208 (1 μM to 10 μM) or DMSO, and cell number was assessed over a period of six days. Interestingly, Raji and Ramos cells were mostly resistant to the drug (Fig. 1a), with a moderate inhibition of cell growth seen in Ramos cells at 10 μM AZD1208 only. Similarly, OCI-Ly3 cells showed a moderate reduction in cell number at 5 μM and 10 μM AZD1208 and only OCI-Ly10 cells displayed a reduced cell number already at 1 μM AZD1208 (Fig. 1a). Nevertheless, PIM kinases were already strongly inhibited in all cell lines at 1 μM AZD1208, as assessed by BAD-S112 phosphorylation (p-BAD) (Fig. 1b, c). This observation suggests that PIM kinase activity is not required for proliferation and survival of these NHL cells. An upregulation or stabilisation of BAD was seen in Raji, OCI-Ly3 and OCI-Ly10 cells at higher AZD1208 concentrations (Fig. 1b, c). Moreover, a compensatory upregulation or stabilisation of PIM1 and PIM2 could be observed in all four cell lines (Fig. 1b, d), suggesting a mechanism compensating for the loss of PIM activity. C-MYC protein levels were also elevated in inhibitor-treated compared to DMSO control cells (Fig. 1b, d). In summary, ABC-DLBCL cell lines showed modest sensitivity to PIM kinase inhibition and both c-MYC-dependent BL cell lines were able to grow independently of PIM kinase activity. This suggests that PIM kinase activity might not be essential for c-MYC-dependent transcription in these cells.

In order to confirm the limited impact of repressing PIM kinases on NHL cell proliferation, two structurally similar kinase inhibitors, SMI4a, and PIM1/2 inhibitor VI (inh VI), and another structurally distinct inhibitor, Quercetagetin, were tested in the same cell lines (Additional file 1: Figures S2, S3). When cells were treated with low doses of these compounds, the anti-proliferative effect was limited (data not shown). Therefore, subsequent experiments were performed with 40 μM of the drugs. In all cell lines, SMI4a caused a marked reduction in cell number and viability (Additional file 1: Figure S2). Quercetagetin and inh VI also repressed cell proliferation with various efficiency in the NHL cell lines tested (Additional file 1: Figures S3A, S3B). As a control, PIM1/2-low SUDHL6 and OCI-Ly19 cells, two GCB-DLBCL-derived cell lines, were also treated with all three inhibitors. The cell number was reduced in inhibitor-treated SUDHL6 and OCI-Ly19 cells compared to DMSO-treated cells on days 3 and 4. Interestingly, Quercetagetin was very toxic for OCI-Ly19 cells, although PIM1 and PIM2 were undetectable in these cells (Additional file 1: Figures S1A, S3C). Altogether, the high inhibitor concentration (40 μM) necessary to observe any significant reduction in cell proliferation and the overall lack of consistency in response to treatments between the cell lines tested, suggest that the anti-proliferative effect of these molecules might be an off-target effect independent of PIM kinase inhibition.

### Knockdowns of c-MYC, PIM1 or PIM2 differentially affect growth of Raji cells

**Fig. 2:**
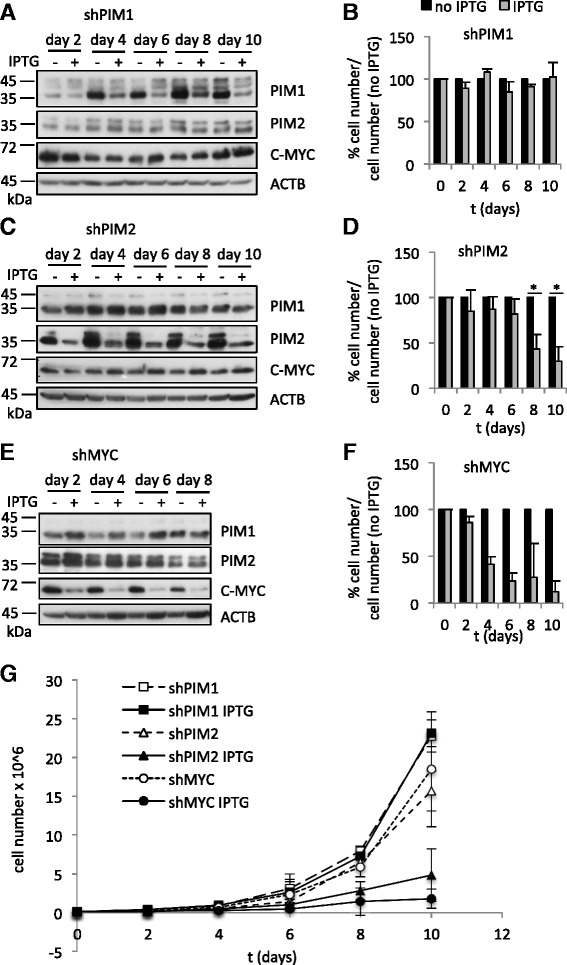
PIM2 knockdown represses proliferation of Raji cells. **a**, **c**, **e** Western blots showing knockdown of PIM1, PIM2 and c-MYC after treatment with 5 mM IPTG in one representative clone each. In absence of IPTG, the different shRNAs were not expressed. **b**, **d**, **f** Cell numbers, as determined by MTT assay, of untreated and IPTG-treated cells were normalised to untreated cells. The means and SD of n independent experiments conducted in duplicate wells of six-well plates are shown. *n* = 3 for shPIM1, *n* = 3 for shPIM2, *n* = 2 for shMYC. **g** Means and SD of absolute cell numbers from (**b**, **d** and **f**) are plotted

To further analyse the role of PIM kinases in mediating proliferation and survival of NHL cells, validated shRNAs against *PIM1*, *PIM2* and c-*MYC*, cloned into the pLKO_IPTG_3xLacO expression vector, were stably transfected into Raji cells and single cell clones were generated. shRNA expression was induced with 5 mM IPTG and the effect of PIM1, PIM2 or c-MYC knockdown on cell viability, cell number and protein expression of IPTG-treated cells was assessed for up to ten days post IPTG treatment. C-MYC knockdown was very efficient (Fig. 2e) and consistent with the established obligatory dependence of BL cells on c-MYC function, resulted in a rapid loss of cell viability compared to untreated cells (Fig. 2f, g). This confirmed that the Raji cell system provided a suitable model in which to test co-modulators of c-MYC function. For PIM1, knockdown of protein expression was seen in IPTG-treated cells from day 4 onwards, while expression of PIM2 and c-MYC remained unchanged confirming specificity (Fig. 2a). However, there was no effect on cell viability when knockdown cells were compared to their untreated counterpart (Fig. 2b, g). Therefore, PIM1 is either dispensable for Raji cell viability or the residual low amount of PIM1 is sufficient to fulfil its essential cellular functions. Knockdown of PIM2 was more efficient than PIM1 knockdown and occurred from day 2 onwards (Fig. 2c). Interestingly, a moderate upregulation of c-MYC was seen after knockdown. Cell viability was significantly reduced on day 8 and day 10 in these cells (Fig. 2d, g). These findings suggest that, in Raji cells, PIM2 plays a more important role than PIM1 in maintaining cell viability, and possibly c-MYC-function. Alternatively, a dose effect could explain these observations, with cell viability being affected when the general PIM kinase level fall below a certain threshold, independently of the targeted PIM.

### Combining AZD1208 treatment and PIM2 knockdown synergistically affects Raji cell growth

**Fig. 3:**
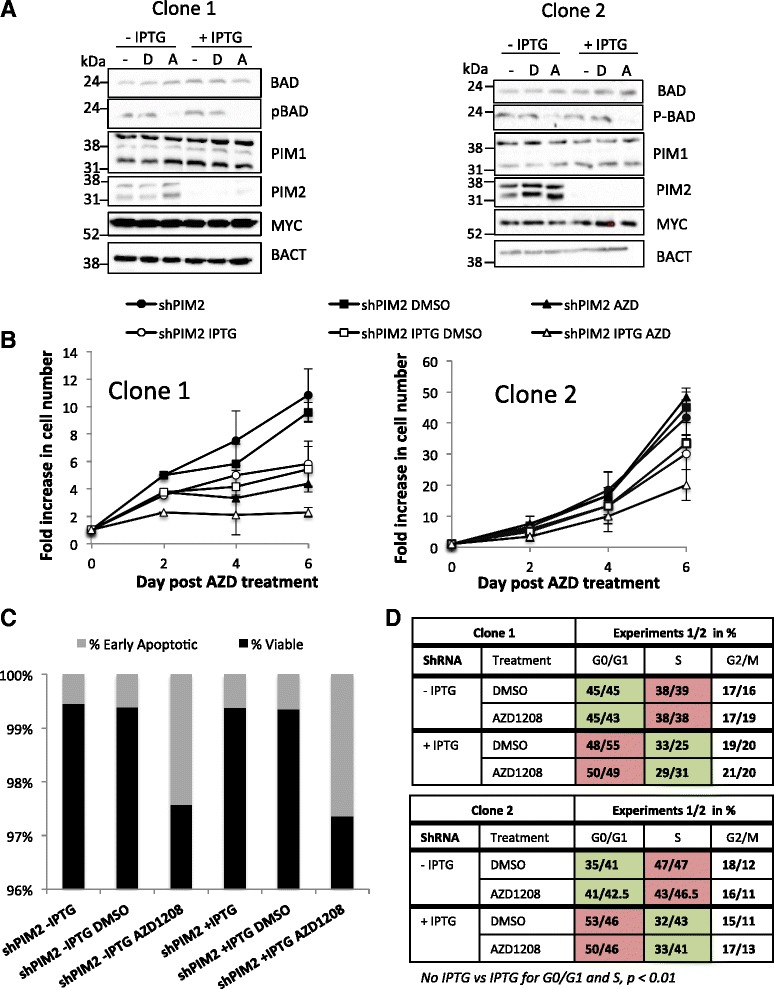
Both AZD1208 treatment and PIM2 knockdown act additively to block Raji cells proliferation. **a**-**d** Cells from two different single cell clones expressing shPIM2 were incubated with or without 5 mM IPTG for two days before drug treatment and then maintained throughout with 5 μM AZD1208 or DMSO used as vehicle control. In absence of IPTG, shPIM2 was not expressed. Media was changed and fresh drug/IPTG added every 2 days. **a** Western blots showing knockdown of PIM2 at day 6, after treatment with or without 5 mM IPTG and DMSO (**d**) or AZD1208 (**a**). **b** Viable cell counts using trypan blue. The means and SD of 2 independent experiments conducted in triplicate wells of six-well plates are shown. **c** Analysis of clone 1 cell viability at day 6, using Annexin V and propidium iodide (PI) staining as measured by flow cytometry and expressed in % viable and apoptotic cells. No change in the percentage of apoptotic cells is observed with clone 2. **d** Cell cycle analysis: cellular DNA was stained with PI and the percentage of cells in G0/G1, S and G2/M phases measured by flow cytometry. For each clone, the experiment has been repeated twice. Paired student’s *t* test comparing no IPTG versus IPTG is indicated at the bottom of the figure. This *t* test is not significant when comparing DMSO and AZD1208 treatments

To further analyse the impact of PIM kinases on the proliferative potential of BL-derived cell lines, we cultured two individual shPIM2 clones with a low (clone 1) or high (clone 2) proliferation rate. The two clones were incubated with or without IPTG for 48 h, then AZD1208 or DMSO was added for up to 6 days. In absence of the pan-PIM kinase inhibitor, the PIM2 knockdown reduced cell proliferation by 30 % (clone 2) and 45 % (clone 1) on day 6 (Fig. 3a, b), which is in agreement with the results previously observed (Fig. 2). When treated with AZD1208 in the absence of IPTG, clone 1 was very sensitive to AZD1208 treatment with almost 60 % reduction in cell proliferation, whereas clone 2 was resistant (Fig. 3b). However, when these cells where treated with both the pan-PIM kinase inhibitor and IPTG, cell proliferation decreased by approximately 55 % (clone 2) to 80 % (clone 1) indicating an additive effect between both approaches to targeting PIM kinases (Fig. 3b). Importantly, while AZD1208 induced apoptosis, PIM2 knockdown was mainly associated with an alteration of the cell cycle (Fig. 3c, d). Compared to untreated cells, PIM2 knockdown resulted in a 15 to 20 % decrease in cells in S phase and an 8 to 22 % increase in cells in G0/G1 phase, independently of the presence of the pan-PIM kinase inhibitor (Fig. 3d). These different effects of AZD1208 and PIM2 knockdown argue for a kinase-independent role of PIM2 in regulating cell growth in Raji cells.

### AZD1208 and PIM2 knockdown differentially alter histone H3K9acS10p at a c-MYC/ PIM1-bound promoter

**Fig. 4:**
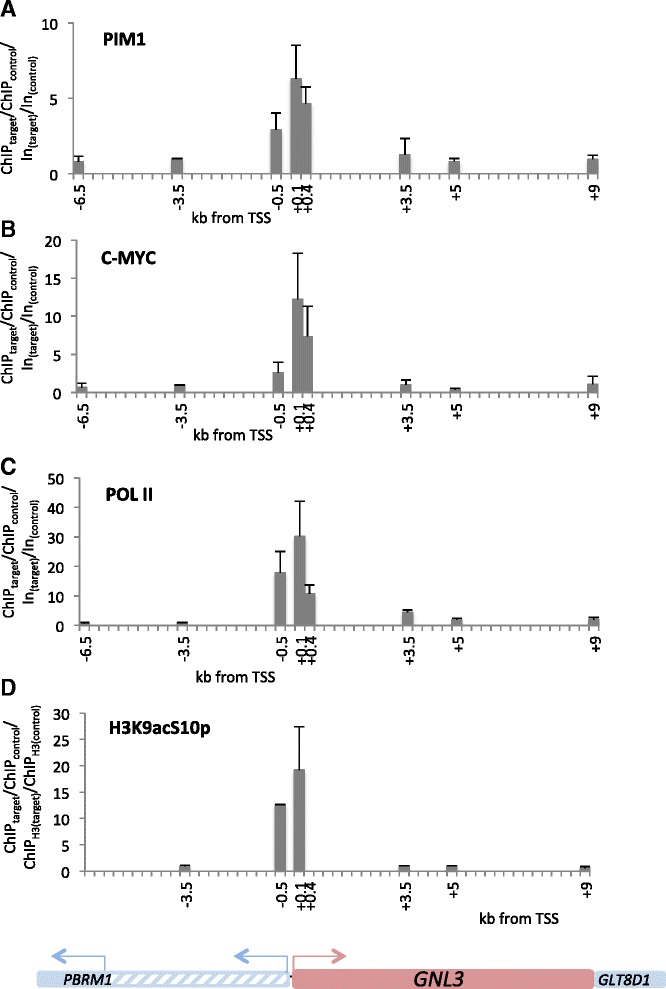
PIM1 and c-MYC bind to the *GNL3* promoter. ChIP was carried out using chromatin from Raji cells and antibodies against PIM1 (**a**), c-MYC (**b**), Pol II CTD (**c**) or H3K9acS10p (**d**). Eluted gDNA was then subjected to qPCR using primer pairs in different regions of the *GNL3* gene. **a** qPCR results were normalised to input and the *GNL3* -3.5 kb region. Means and SD of six independent experiments are shown. **b** Results are shown normalised to input and the *GNL3* -3.5 kb region. Means and sd of five independent experiments are shown for Raji cells, whereas one experiment was carried out in OCI-Ly10 cells. **c** Results are again normalised to input and the *GNL3* -3.5 kb region. Means and SD of two independent experiments are shown for Raji cells, one experiment was done in OCI-Ly10 cells. **d** Results are normalised to H3 and the GNL3 -3.5 kb region. The means and SD of three independent experiments are shown

Since PIM1 has been shown to promote c-MYC-dependent transcriptional activity [34], we sought to identify possible PIM1 and c-MYC target genes in lymphoma cell lines. The DLBCL mRNA expression profile generated by Care et al. identified *PIM1* and *PIM2* among the top 20 genes associated with ABC-DLBCL [10]. Therefore, it seemed plausible that other genes associated with the ABC profile in this study might be transcriptionally regulated by PIM kinases. For further analyses, we focused on the known oncogene guanine nucleotide binding protein-like 3 (*GNL3*) and the methionine sulfoxide reductase B1 gene (*MSRB1*) also known as *SEPX1*, which have known E box elements in their promoters. First, ChIP-qPCR was used to identify PIM1 and c-MYC binding sites in these target genes. The known E box elements in *GNL3* (*GNL3* + 0.1 kb, *GNL3* + 0.4 kb) and *SEPX1* (*SEPX1* -0.3 kb) were tested. Further, Zippo et al. described regions upstream of the *ID2* (*ID2* -1.4 kb, *ID2* -1.7 kb) and within the *FOSL1* gene (*FOSL1* + 1.15 kb), which are occupied by PIM1 and c-MYC in HEK293 cells [32]. These were also assessed in the present study. The *FOSL1* -37 kb region served as a negative and *NPM1* + 1 kb served as a positive control, as it is a known MYC-bound site in BL cell lines [50]. ChIP-qPCR showed that PIM1 and c-MYC were bound to the *GNL3* promoter and the *NPM1* intronic enhancer in both Raji and Ramos cells (Fig. 4a, b, Additional file 1: Figure S4). No binding was observed at any of the other regions tested in Raji cells (Additional file 1: Figure S4). The *GNL3* promoter and *NPM1* enhancer regions were also occupied by POL II in both cell lines, indicating that these genes are either actively transcribed or poised for activation (Fig. 4c, Additional file 1: Figure S4). Moreover, POL II was detected at the *SEPX1* promoter (Additional file 1: Figure S4). As expected, phosphorylation of the known PIM1 target site, H3S10, was seen around the *GNL3* promoter.

**Fig. 5:**
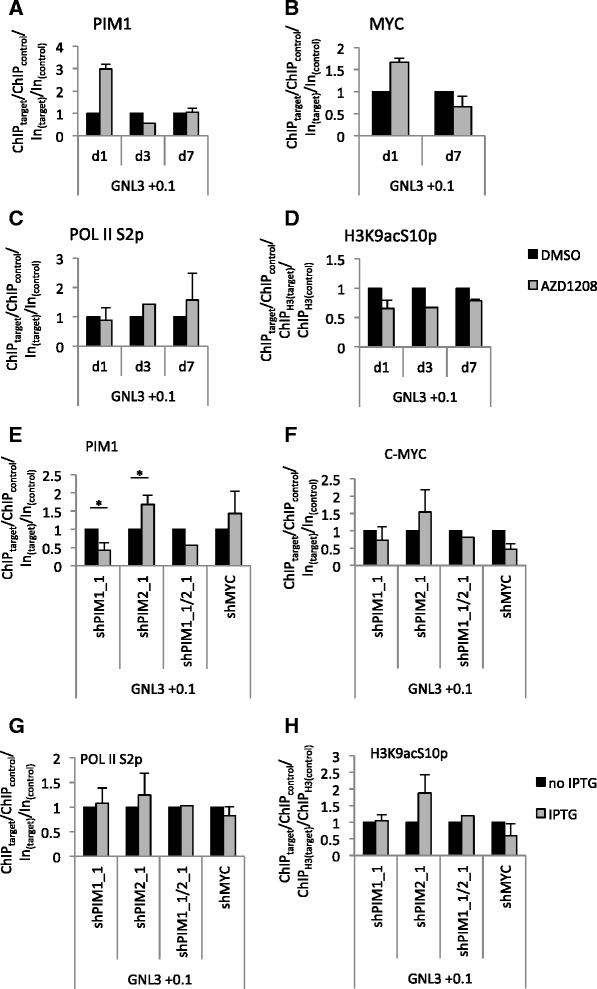
PIM2 knockdown increases PIM1 binding to the *GLN3* promoter. **a**-**d** ChIP was performed in Raji cells after AZD1208 treatment. The cells were treated with 5 μM AZD1208 or DMSO from day 0 for 1, 3 or 7 days. ChIP was done with antibodies against PIM1 (**a**), c-MYC (**b**), H3K9acS10p (**c**) or Pol II S2p (**d**). Results are normalised to input or H3 and control regions (*CTCF1* and *CTCF2* for PIM1, c-MYC and Pol II S2p, *CTCF2* and *GNL3* + 9 for H3K9acS10p) and DMSO-treated cells. The means and SD of 2 independent experiments are shown for days 1 and 7, the result of one experiment is shown for day 3. **e**-**h** ChIP was performed in untreated or IPTG-treated pLKO-shRNA-transfected cells. Antibodies against PIM1 (**e**), c-MYC (**f**), Pol II S2p (**g**) or H3K9acS10p (**h**) were used. The results are normalised to input and control regions (*GNL3* -3.5 and *GNL3* + 9 for PIM1 and c-MYC (**e**, **f**), *CTCF1* and *GNL3* -3.5 for Pol II S2p (**g**) and *CTCF1* for H3K9acS10p (**h**)) and plotted relative to untreated cells. Means and SD of n independent experiments are shown, with *n* = 3 for shPim1, *n* = 3 for shPim2, *n* = 1 for shPim1/2 and *n* = 2 for shcMyc. For *n* = 3 experiments, two-tailed Student’s *t* test was performed: *p < 0.05

Then, we analysed PIM1 and c-MYC recruitment to the *GNL3* cis-element in Raji cells following PIM kinase inhibitor treatment. Cells were treated with 5 μM AZD1208 for one, three or seven days. Consistent with effective inhibition of PIM kinase activity, H3K9acS10p was reduced after AZD1208 treatment (Fig. 5d). However, a transient increase in c-MYC and PIM1 binding at the *GNL3* promoter on day 1 was observed which was not sustained on day 3 and 7 (Fig. 5a, b). This correlated with the increased c-MYC and PIM1 protein levels in AZD-treated cells (Fig. 1b) and suggests that PIM kinase activity is required for turnover at the *GNL3* promoter. Surprisingly, however, no significant change in the recruitment of the elongating POL II S2p could be seen (Fig. 5c), suggesting that H3S10p is not required for *GNL3* active elongation. In agreement, transcript levels of *GNL3* were unaltered (data not shown).

ChIP experiments confirmed that PIM1 binding to the *GNL3* promoter was significantly reduced after knockdown compared to non-IPTG-treated cells (Fig. 5e). Interestingly, PIM2 knockdown led to an increase in PIM1 binding to DNA, suggesting that PIM2 might interfere with PIM1 recruitment or affect PIM1-binding turnover (Fig. 5e). As expected, PIM1 binding to the *GNL3* promoter also decreased after PIM1/2 double knockdown. Surprisingly, c-MYC knockdown had no effect on the presence of PIM1 at the *GNL3* + 0.1 kb site, although the presence of c-MYC was reduced in this region (Fig. 5e, f). This suggests, that PIM1 can occupy cis-regulatory elements independently of c-MYC. In the reciprocal experiment, knockdown of the PIM kinases did not alter c-MYC occupancy at the *GNL3* promoter (Fig. 5f). No change in H3K9acS10p was seen in PIM1 or PIM1/2 knockdown cells, while there was an increase after PIM2 knockdown, consistent with the increased binding of PIM1, and a decrease in c-MYC knockdown cells (Fig. 5h). Given that AZD1208 treatment reduced H3K9acS10p, while knockdown of PIM1 and PIM2 did not, another AZD1208 sensitive kinase may substitute for H3S10 phosphorylation in the absence of PIM1/2.

## Discussion

In this study, the potent and selective PIM kinase inhibitor AZD1208 did not significantly reduce proliferation of BL or ABC-DLBLC cell lines. AZD1208 has been extensively tested against a panel of 442 kinases and inhibited only 13 kinases other than PIM1/2/3 by 50 % or more, but was still at least 43-fold selective for PIM kinases [51]. Furthermore, in the study by Keeton et al., AZD1208-sensitive cell lines showed increased apoptosis already at 1 μM AZD1208 [51], a concentration at which none of the cell lines tested in this study displayed reduced viability. Nevertheless, AZD1208 efficiently inhibited PIM kinases, as evaluated by BAD-S112 phosphorylation, at a concentration of 1 μM in the two BL and the two ABC-DLBCL cell lines assessed in our study. In agreement with a previous study, in which pan-PIM kinase inhibition led to stabilisation of PIM3 [52], efficient inhibition of PIM kinases also led to a stabilisation of PIM1 and PIM2. In contrast, SMI4a, inh VI and Quercetagetin had a significant impact on cell proliferation in the same cell lines. However, their anti-proliferative potential was observed at high concentration and was heterogeneous between the different cell lines suggesting an effect independent of PIM kinase inhibition. Consequently, these data argue for a limited role of PIM kinase activity in maintaining oncogenicity in NHL and are consistent with previous findings [11]. In agreement with this conclusion, knockdown of PIM1 had no significant impact on Raji cell proliferation. In addition, while AZD1208 treatment correlated with a decrease in H3K9acS10p at the *GNL3* promoter, this histone posttranslational modification was not significantly reduced by c-MYC or PIM kinase knockdown, suggesting that another kinase may target H3S10 at the *GNL3* promoter, at least in absence of PIM1. Several kinases have been shown to target H3S10 [53] and a certain level of redundancy between all these enzymes might exist, which would preserve a normal gene expression programme if the activity of one of them was altered.

In contrast, PIM2 knockdown significantly reduced Raji cell number, suggesting that PIM2 might have an important function in maintaining Burkitt lymphoma cell growth. This is consistent with PIM2 being more frequently overexpressed in different haematological malignancies than PIM1 and more significantly associated with the activation of oncogenic pathways [12]. On the other hand, the knockdown of PIM2 was consistently more efficient than that of PIM1, and it cannot be excluded that PIM1 depletion in this study might not have been sufficient to alter the proliferative capacity of NHL cell lines. Because pan-PIM kinase inhibition did not significantly reduce cell proliferation, although it clearly abrogated PIM activity as assessed by BAD phosphorylation, it can be hypothesised that PIM2 might have kinase-independent functions in DLBCL and BL. A function of PIM, independent of its kinase activity, has already been described for PIM1. First, overexpression of kinase-dead PIM1 can mimic some functions of active PIM1 [54, 55]. Furthermore, PIM1 is recurrently targeted by aberrant somatic hypermutation in DLBCL, but out of 5 mutant proteins analysed, only one showed increased kinase activity, while three mutants were significantly less active than the wildtype protein [56]. Altogether, these previous observations and our current work suggest that PIM kinases have kinase-independent functions in lymphomagenesis.

In agreement with a role of PIM2 in cell proliferation independent of its kinase activity, a combination of AZD1208 treatment and PIM2 knockdown additively repressed proliferation of Raji cells for clones either sensitive or resistant to AZD1208 treatment only. In this context, AZD1208 was associated with a reduction in cell viability, whereas PIM2 knockdown altered cell cycle progression. Several studies have pointed to a function of c-MYC in DNA replication licensing, c-MYC being known to control DNA replication by direct interaction with the pre-replication complex [57]. Because, none of the inhibitor or knockdown experiments conducted in this study had any impact on *GNL3* gene expression, the presence of c-MYC and PIM1 at the *GNL3* promoter might participate in c-MYC-associated DNA replication licensing. Interestingly, repression of S-phase entry after PIM2 knockdown is in agreement with a defect in DNA replication licensing and coincides with an increase in PIM1 recruitment to the *GNL3* gene. This enhanced recruitment might be a consequence of direct competition for protein-protein interaction with c-MYC, or might indicate other interactions between PIM1 and PIM2 important for PIM1 function. PIM2 could, for example, inhibit nuclear translocation of PIM1 or inhibit association of PIM1 with chromatin. Nevertheless, this inverted correlation between cell proliferation and PIM1 enrichment at the c-MYC-bound GNL3 promoter suggests a repressive role of the chromatin-associated PIM1 in cell cycle progression, independent of its kinase activity. For example, the presence of this kinase might prevent the recruitment of another complex at c-MYC-bound cis-regulatory elements. Further investigations, beyond the scope of this work will be necessary to clarify this observation.

## Conclusions

In conclusion, our data show that PIM kinase inhibition has a limited impact on NHL cell growth, possibly due to the fact that this inhibition does not completely abolish PIM function and a kinase-independent role of PIM kinases in cell cycle regulation.

## Methods

### Cell culture

OCI-Ly3 and OCI-Ly10 cells (kind gift of Prof. R.E. Davies) were maintained in IMDM substituted with 20 % Foetal Calf Serum (FCS) and Penicillin-Streptomycin (pen/strep). OCI-Ly19, SUDHL6, Raji and Ramos cells (ATCC) were maintained in RPMI-1640 with 10 % FCS and pen/strep. Treatment of cells with PIM kinase inhibitors was done with 40 μM SMI4a (Enzo Life Sciences), 40 μM Quercetagetin (Merck Millipore), 40 μM PIM1/2 inhibitor VI (Merck Millipore), 1 to 10 μM AZD1208 (Active Biochemicals) or DMSO (control). The medium was changed every second day and cell proliferation was measured using an MTT assay (Sigma-Aldrich).

### Flow cytometry

Apoptosis was assessed using the Annexin V Apoptosis Detection Kit with propidium iodide (PI) (Biolegend Pacific Blue™) according to the manufacturer’s recommendations. For cell cycle analysis, DNA was stained with PI and cells were analysed using a BD LSRII flow cytometer. The percentage of cells in each stage of the cell cycle was then determined using the ModFit software (Verity Software House).

### Generation of stably transfected Raji cell lines

The pLKO_IPTG_3xLacO-shLuc vector was purchased from Sigma Aldrich and shMYC, shPIM1 and shPIM2 were cloned into this vector (for shRNA sequences see Additional file 2: Table S1). Raji cells were transfected using the Amaxa™ Nucleofector™ as indicated by the manufacturer. Briefly, 10^7^ cells and 10 μg DNA were suspended in 100 μl Nucleofector Solution V and transfected using programme M-013. Stable cells were selected with 1 μg/ml puromycin and single cell clones were generated. For experiments, stably transfected Raji cells were seeded into appropriate tissue culture dishes and left untreated or were treated with 5 mM IPTG in normal culture medium. The medium with IPTG was renewed every second day and the cells were counted or RNA, protein or chromatin were harvested after two to ten days.

### Western blots

Cells were lysed in RIPA buffer (10 mM Tris–HCl, 1 mM EDTA, 0.5 mM EGTA, 140 mM NaCl, 0.1 % SDS, 1 % Triton X-100, 0.1 % Na Deoxycholate, proteinase inhibitor cocktail (P8340, Sigma Aldrich), 2 μM PMSF, 1 mM DTT, 0.5 mM NaF, 2 mM NaVO_3_) and protein concentrations were determined using the Bradford assay (Bio-Rad). Western blots were carried out under denaturing conditions with SDS-PAGE, proteins were transferred to PVDF membranes (for blocking conditions and primary antibody concentrations see Additional file 2: Table S2). HRP conjugated secondary antibodies were used at 1:10,000 (eBioscience).

### Chromatin immunoprecipitation assays and real-time PCR analysis

Chromatin immunoprecipitation was performed as previously described [58] using 10 μl dynabeads protein G (Invitrogen) with 2.4 μg of anti-POL II (Abcam, ab817), anti-histone H3 (Abcam, ab1791), anti-H3K9acS10p (Abcam, ab12181), anti-c-MYC (Santa Cruz Biotechnology, sc-764), anti-PIM1 (Bethyl Laboratories, A300-313A) and anti-RNA POL II S2p (Abcam, ab5095) antibodies. For ChIP primer sequences see Additional file 2: Table 1.

## Additional files

Additional file 1: Figure S1. PIM1 and PIM2 expression in NHL cell lines. A Protein expression of PIM kinases was assessed in different BCL cell lines by western blot after nuclear-cytoplasmic fractionation. The data are representative of three independent experiments. B mRNA expression of PIM1 and PIM2 was measured by RT-qPCR and is shown relative to *TBP* expression. Means and sd of two to five independent experiments are plotted. **Figure S2:** Smi4a represses proliferation of NHL cell lines. (A) Raji, (B) Ramos, (C) OCI-Ly10 and (D) OCI-Ly3 were treated with 40 μM SMI4a starting from day 0. Cell number was assessed by MTT assay. Left: One experiment is shown for each cell line. Right: The means and SD of at least two independent experiments conducted in duplicate wells of a 6-well plate are plotted. For n ≥ 3 Student’s *t* test was performed: **p* < 0.05, ***p* < 0.01. **Figure S3:** Anti-proliferative potential of various PIM kinase inhibitors on NHL cell lines. Burkitt lymphoma Raji and Ramos (A), ABC-DLBCL OCI-Ly3 and OCI-Ly10 (B) and GCB-DLBCL SUDHL6 and OCI-Ly19 cells (C) were treated with 40 μM Quercetagetin or PIM1/2 inhibitor VI or SMI4a (SUDHL6, OCI-Ly19) for indicated times. Treatment started on day 0 and cells were counted daily using Trypan blue staining and a haemocytometer. The averages of one experiment conducted in duplicate wells are shown. For DMSO averages and standard deviations of two independent experiments are plotted. **Figure S4:** Identification of c-MYC/PIM1-bound cis-regulatory elements by ChIP in BL-derived cell lines. ChIP for PIM1, c-MYC and Pol II was done in Raji and Ramos cells and primers for different possible c-MYC binding sites were used for qPCR. For Raji cells, means and standard deviations of two technical replicates are shown, values for Ramos cells are from one experiment. PIM1 and c-MYC binding was observed at the *GNL3* promoter (+0.1kb, +0.4kb from the TSS) and the *NPM1* enhancer (+1kb from the TSS) in both cell lines. The *ID2*, *SEPX1* and *FOSL1* sites were negative for PIM1 and c-MYC in Raji cells, but binding at the *ID2* -1.4kb region and the *FOSL1* gene was observed in Ramos cells. POL II was enriched at the *GNL3* promoter and *NPM1* enhancer, but also at the *SEPX1* promoter in both cell lines. The other tested regions were showed only very low Pol II binding. (PDF 1050 kb) Additional file 2: Table S1. Designed primers and ShRNAs. **Table S2:** Western Blotting antibodies. (PDF 102 kb)
