# Supplementary material for: Loss of PIM2 enhances the anti-proliferative effect of the pan-PIM kinase inhibitor AZD1208 in non-Hodgkin lymphomas
Source: Mol Cancer. 2015 Dec 8;14:205. doi: 10.1186/s12943-015-0477-z (PMC4672512; doi:10.1186/s12943-015-0477-z)
Supplement: Additional file 1: Figure S1. — PIM1 and PIM2 expression in NHL cell lines. A Protein expression of PIM kinases was assessed in different BCL cell lines by western blot after nuclear-cytoplasmic fractionation. The data are representative of three independent experiments. B mRNA expression of PIM1 and PIM2 was measured by RT-qPCR and is shown relative to TBP expression. Means and sd of two to five independent experiments are plotted. Figure S2: Smi4a represses proliferation of NHL cell lines. (A) Raji, (B) Ramos, (C) OCI-Ly10 and (D) OCI-Ly3 were treated with 40 μM SMI4a starting from day 0. Cell number was assessed by MTT assay. Left: One experiment is shown for each cell line. Right: The means and SD of at least two independent experiments conducted in duplicate wells of a 6-well plate are plotted. For n ≥ 3 Student’s t test was performed: *p < 0.05, **p < 0.01. Figure S3: Anti-proliferative potential of various PIM kinase inhibitors on NHL cell lines. Burkitt lymphoma Raji and Ramos (A), ABC-DLBCL OCI-Ly3 and OCI-Ly10 (B) and GCB-DLBCL SUDHL6 and OCI-Ly19 cells (C) were treated with 40 μM Quercetagetin or PIM1/2 inhibitor VI or SMI4a (SUDHL6, OCI-Ly19) for indicated times. Treatment started on day 0 and cells were counted daily using Trypan blue staining and a haemocytometer. The averages of one experiment conducted in duplicate wells are shown. For DMSO averages and standard deviations of two independent experiments are plotted. Figure S4: Identification of c-MYC/PIM1-bound cis-regulatory elements by ChIP in BL-derived cell lines. ChIP for PIM1, c-MYC and Pol II was done in Raji and Ramos cells and primers for different possible c-MYC binding sites were used for qPCR. For Raji cells, means and standard deviations of two technical replicates are shown, values for Ramos cells are from one experiment. PIM1 and c-MYC binding was observed at the GNL3 promoter (+0.1kb, +0.4kb from the TSS) and the NPM1 enhancer (+1kb from the TSS) in both cell lines. The ID2, SEPX1 and FOSL1 sites were negative for P [file 12943_2015_477_MOESM1_ESM.pdf]

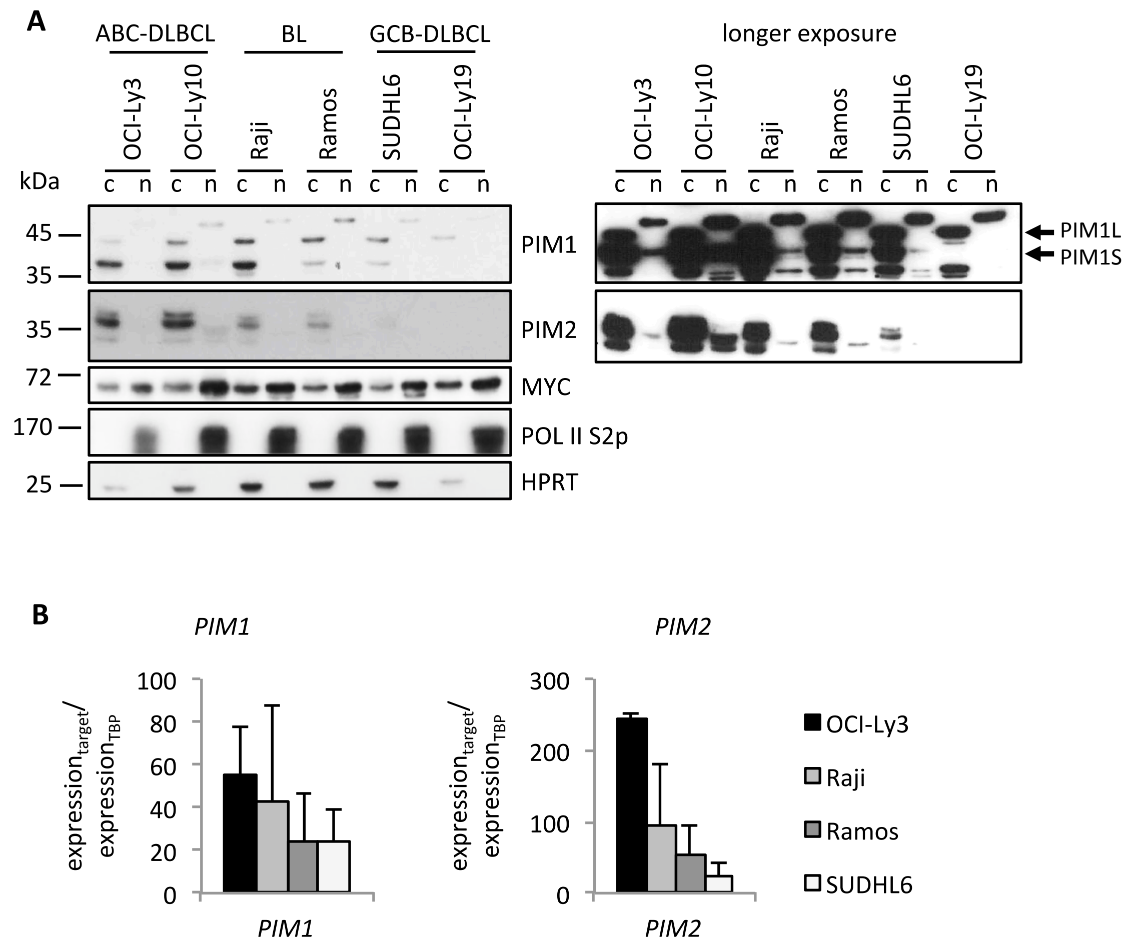

Figure S1: PIM1 and PIM2 expression in NHL cell lines. A Protein expression of PIM kinases was assessed in different BCL cell lines by western blot after nuclear-cytoplasmic fractionation. The data are representative of three independent experiments. **B** mRNA expression of PIM1 and PIM2 was measured by RT-qPCR and is shown relative to *TBP* expression. Means and sd of two to five independent experiments are plotted.

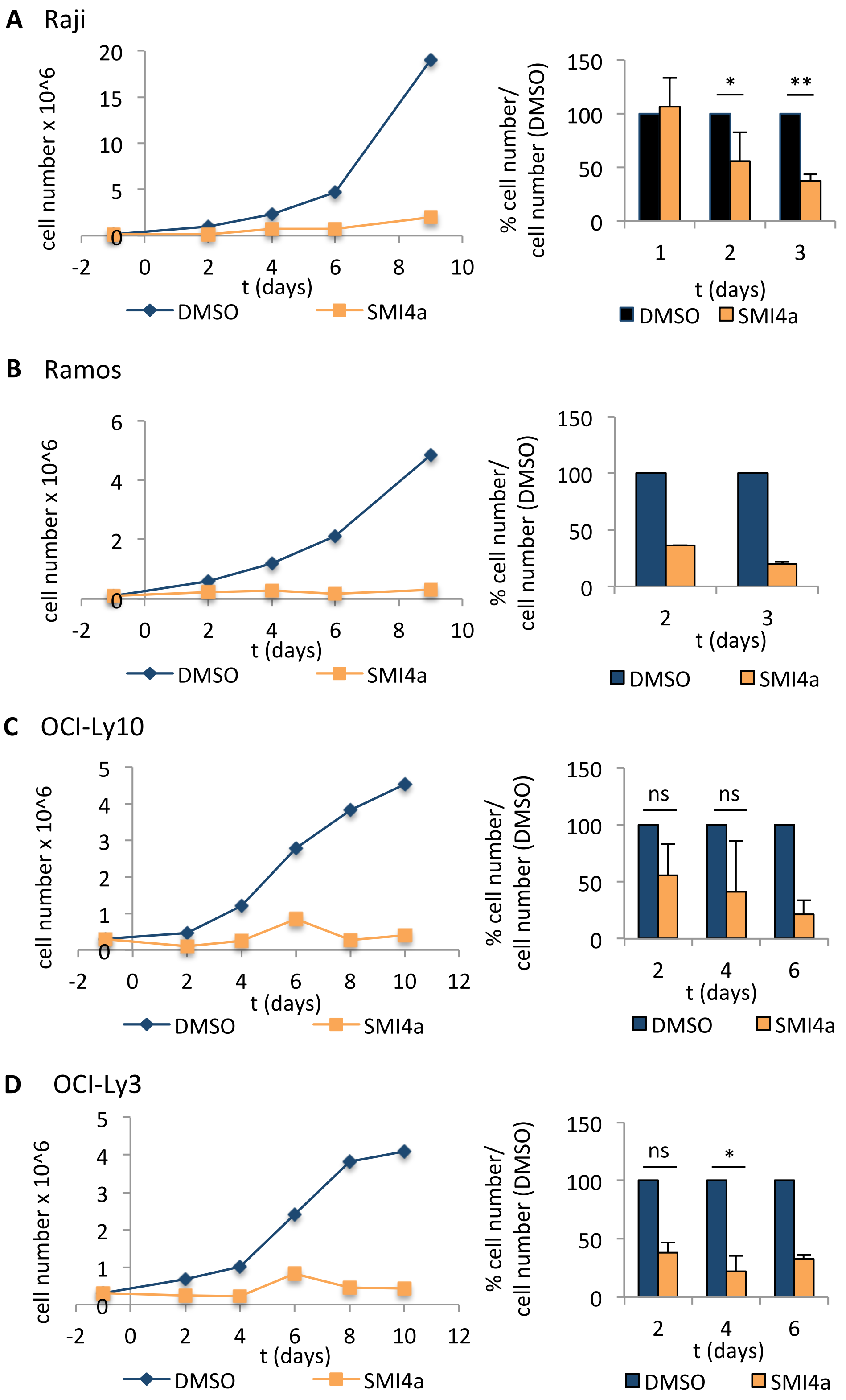

Figure S2: Smi4a represses proliferation of NHL cell lines. (**A**) Raji, (**B**) Ramos, (**C**) OCI-Ly10 and (**D**) OCI-Ly3 were treated with 40 μM SMI4a starting from day 0. Cell number was assessed by MTT assay. Left: One experiment is shown for each cell line. Right: The means and SD of at least two independent experiments conducted in duplicate wells of a 6-well plate are plotted. For n ≥ 3 Student's t test was performed: * $p < 0.05$, ** $p < 0.01$.

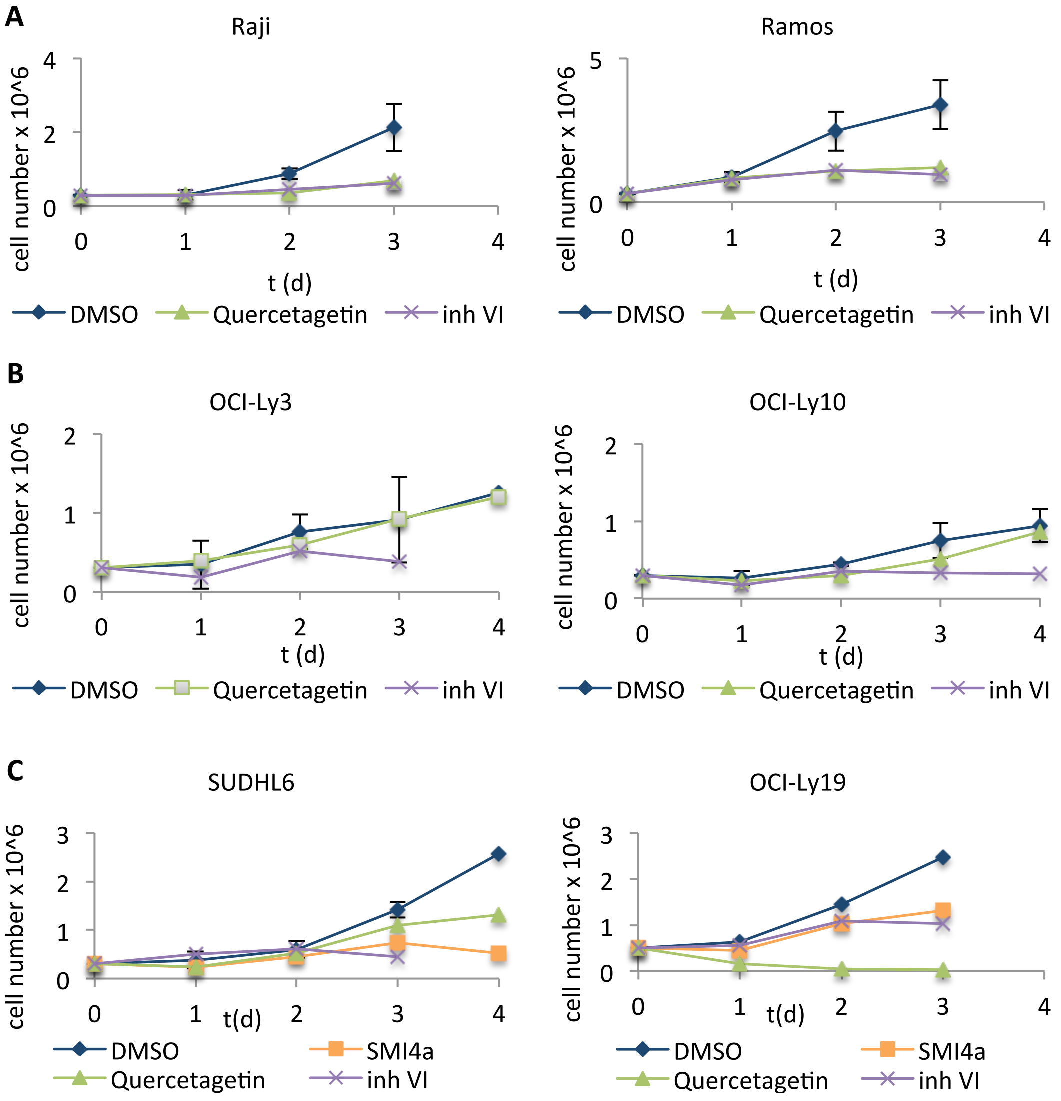

Figure S3: Anti-proliferative potential of various PIM kinase inhibitors on NHL cell lines. Burkitt lymphoma Raji and Ramos (**A**), ABC-DLBCL OCI-Ly3 and OCI-Ly10 (**B**) and GCB-DLBCL SUDHL6 and OCI-Ly19 cells (**C**) were treated with 40 μM Quercetagetin or PIM1/2 inhibitor VI or SMI4a (SUDHL6, OCI-Ly19) for indicated times. Treatment started on day 0 and cells were counted daily using Trypan blue staining and a haemocytometer. The averages of one experiment conducted in duplicate wells are shown. For DMSO averages and standard deviations of two independent experiments are plotted.

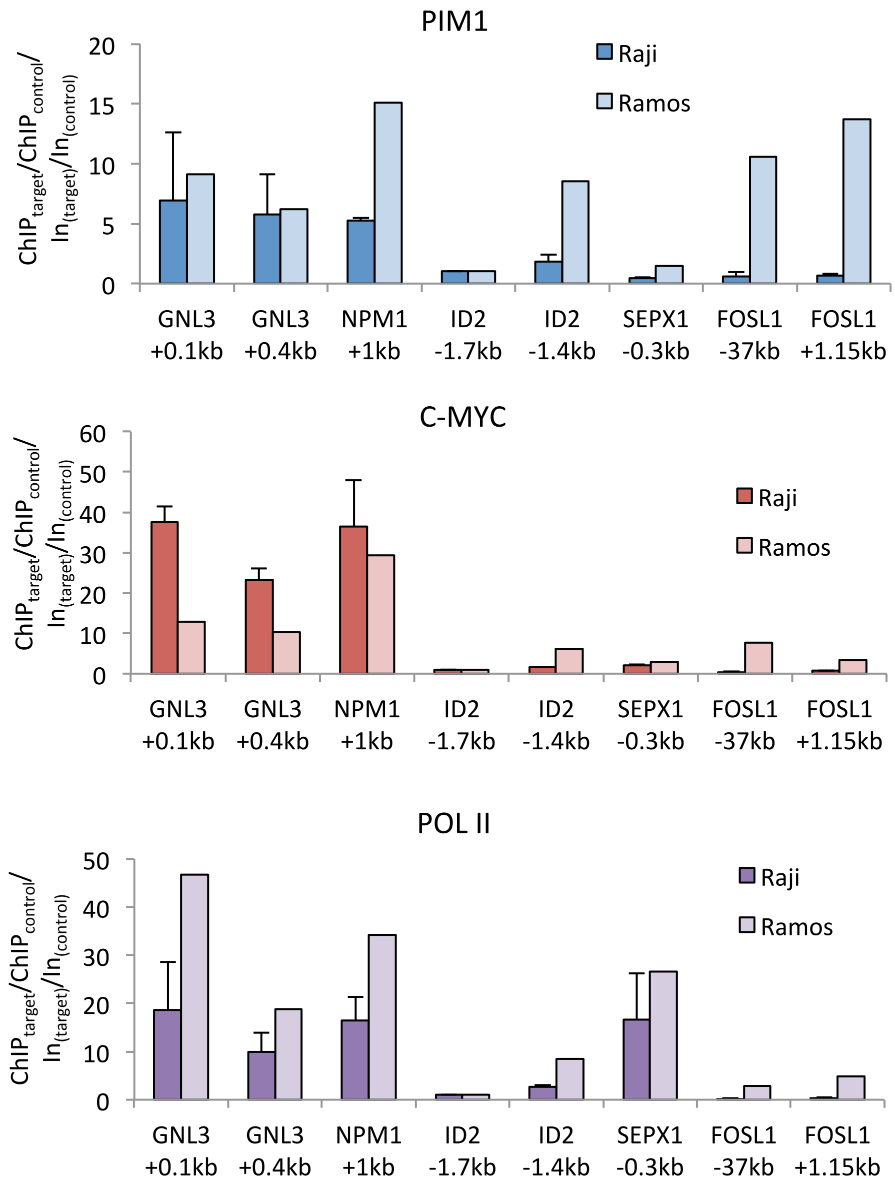

Figure S4: Identification of c-MYC/PIM1-bound cis-regulatory elements by ChIP in BL-derived cell lines. ChIP for PIM1, c-MYC and Pol II was done in Raji and Ramos cells and primers for different possible c-MYC binding sites were used for qPCR. For Raji cells, means and standard deviations of two technical replicates are shown, values for Ramos cells are from one experiment. PIM1 and c-MYC binding was observed at the *GNL3* promoter (+0.1kb, +0.4kb from the TSS) and the *NPM1* enhancer (+1kb from the TSS) in both cell lines. The *ID2*, *SEPX1* and *FOSL1* sites were negative for PIM1 and c-MYC in Raji cells, but binding at the *ID2* -1.4kb region and the *FOSL1* gene was observed in Ramos cells. POL II was enriched at the *GNL3* promoter and *NPM1* enhancer, but also at the *SEPX1* promoter in both cell lines. The other tested regions were showed only very low Pol II binding.
