# Supplementary material for: Loss of PIM2 enhances the anti-proliferative effect of the pan-PIM kinase inhibitor AZD1208 in non-Hodgkin lymphomas
Source: Mol Cancer. 2015 Dec 8;14:205. doi: 10.1186/s12943-015-0477-z (PMC4672512; doi:10.1186/s12943-015-0477-z)
Supplement: Additional file 2: Table S1. — Designed primers and ShRNAs. Table S2: Western Blotting antibodies. (PDF 102 kb) [file 12943_2015_477_MOESM2_ESM.pdf]

Supplementary table 1: Designed primers and ShRNAs

Function	Location	Sequence forward	Sequence reverse
ChIP *GNL3* locus	GNL3 -0.5	AGGCCCTGAGCAACGGAGGAA	GCGCACTACGGGTCGGAAACTC
ChIP *GNL3* locus	GNL3 -3.5	CCTGCCCAACTGCCCCCTAGTC	GGGGGAGGGTGTTTTCCAGACCT
ChIP *GNL3* locus	GNL3 -6.5	TCCTGCTTGGGCCTGGTGTTGA	GCTACCTCCCCTTCCAGCAGTGT
ChIP *GNL3* locus	GNL3 +0.1	CCAGCGGAGGCAGGTTGATGTG	TGGCTGCAGCAACTTCCAGACG
ChIP *GNL3* locus	GNL3 +0.4	TTAACCGCGCGGCTCATTCTG	CAGCTCTCACCTGGGCCAGTCT
ChIP *GNL3* locus	GNL3 +3.5	GCCTCCGATGTTGTCCTAGAGGTGTT	TCCCCACTGCCTAGCACTGTCTC
ChIP *GNL3* locus	GNL3 +5	TGGGCTCTGGAGCCTGATTGCT	AGCATTCTTCTTTGCCTTCACACGCT
ChIP *GNL3* locus	GNL3 +9	CCAAGGTGGCGGACATTCCACA	AGCAGAACCCTGGCTGGTAGCAT
CTCF control	CTCF1	GGCCCAGGACTCCACGTTCAGA	GCCCTCTGGTGTTTGGCAGCAA
CTCF control	CTCF2	CACCCAGCAGAGGGCCCAGATA	CCCTTCGCCTTCTCTCCAGCCA
ChIP *NPM1* gene	NPM1 +1	GCCTGCTTGTTGGAGCGGGTAGA	GCCGAAGCACGCGAGGTAAGTC
ChIP *ID2* locus	ID2 -1.7	AGGCTCGAACTGTGGGAGGACT	CTGCCTGCGACTCTGTCCCTGA
ChIP *ID2* locus	ID2 -1.4	GGAAGGCCCTCCGCAAACTTCT	TGACAGCTATGCGCCCCATGAC
ChIP *SEPX1* pro	SEPX1 -0.3	CGCGGAAAGGCTGCATGACCTC	CGCGAATCCGTAGGGTCGTGTC
ChIP *FOSL1* locus	FOSL1 +1.15	GCCCTCGATCCCTTTGCCGAATG	AGGAAATGGGCACCTGCAGCCT
ChIP *FOSL1* locus	FOSL1 -37	CGGGGATCAAAGATGAAGAA	TGGGGAGCAGATAGCTGAGT
ShRNA	shPIM1	ACATCCTTATCGACCTCAATCCTCGAGGATTGAGGTCGATAAGGATGT	
ShRNA	shPIM2	GATGAACCCTACACTGACTTTCTCGAGAAAGTCAGTGTAGGGTTCATC	
ShRNA	ShMYC	CCTGAGACAGATCAGCAACAACTCGAGTTGTTGCTGATCTGTCTCAGG	

Supplementary table 2: Western Blotting antibodies

Antibody	Catalogue	Company	Block	Primary	Secondary
Anti-H3	ab1791	Abcam	5% Milk	1:100000	1 in 10,000
anti-β-actin	A1978	Sigma Aldrich	5% Milk	1:100000	1 in 10,000
anti-BAD	9239	Cell Signaling Technology	5% Milk	1:1000	1 in 10,000
anti-BADS112p	5284	Cell Signaling Technology	5% BSA	1:1000	1 in 10,000
anti-c-MYC	sc-764	Santa Cruz Biotechnology	5% BSA	1:3000	1 in 10,000
anti-PIM1	2907	Cell Signaling Technology	5% Milk	1:500	1 in 10,000
anti-PIM2	61159	Active Motif	5% BSA	1:3000	1 in 10,000
